# Limiting Performance of Radar-Based Positioning Solutions for the Automotive Scenario

**DOI:** 10.3390/s24247940

**Published:** 2024-12-12

**Authors:** Francesco Bandiera, Giuseppe Ricci

**Affiliations:** Department of Innovation Engineering, University of Salento, 73100 Lecce, Italy; giuseppe.ricci@unisalento.it

**Keywords:** positioning, radar measurements, Cramér–Rao lower bounds

## Abstract

Road safety applications for automotive scenarios rely on the ability to estimate vehicle positions with high precision. Global navigation satellite systems (GNSS) and, in particular, the global positioning system (GPS), are commonly used for self localization. But, especially in urban vehicular scenarios, due to obstructions, they may not provide the requirements for crucial position-based applications. In this paper, we investigate the potential of GPS-free positioning schemes and, in particular, we compute the ultimate performance, i.e., Cramér–Rao lower bounds (CRLB), of localization schemes in which each vehicle estimates its position exploiting range and/or angle measurements of an assigned set of landmarks with a known position.

## 1. Introduction

Road safety applications are emerging as an important feature of intelligent transportation systems (ITS). However, such applications pose numerous challenges for which ultimate solutions are still unavailable. One of the most important issues is how to guarantee accurate position information in the very diverse automotive scenarios [1,2]. Global positioning systems (GPS) are widely used for localization; however, as recent studies [3] show, the accuracy and availability of the GPS signal cannot always meet the requirements of crucial position-based applications. For instance, in dense urban environments, the accuracy and availability of the GPS are limited by satellite visibility interruption, vehicle dynamics, and local errors (e.g., receiver noise and multipath) [4]. Preliminary research efforts, e.g., [5,6,7], have tackled this problem by focusing on standalone positioning systems that combine GPS data with additional measurements gathered from kinematic sensors available on board (Dead Reckoning, INS, etc.).

In recent years, vehicular ad hoc networks (VANETs) [8] have been proposed by the automotive research community as a means to realize a connected road environment where vehicles and infrastructure components can communicate to improve their location awareness [9,10,11,12]. In a VANET, GPS-free positioning techniques can take advantage of the beacon packets transmitted from roadside units (RSUs), possibly in a cooperative fashion by jointly processing position-related data exchanged among a group of VANET nodes [13]. A receiver can exploit either range measurements based on the received signal strength (RSS), time of arrival (TOA), and time difference of arrival (TDOA) [9,14,15] or angle of arrival (AOA) measurements [16,17,18,19] associated with the signals transmitted from nearby anchors to determine its own position. Specifically, in [17] the authors investigate a vehicle-to-infrastructure (V2I) scenario where a vehicle equipped with an array of antennas is able to determine its position processing several AOA estimates collected by the vehicle along its trajectory; AOAs are obtained by the multiple signal classification (MUSIC) algorithm on the basis of packets broadcast by a road-side unit (RSU) in a known position while the trajectory is reconstructed resorting to local INS measurements performed by the vehicle. This approach turns out to outperform GPS in urban environments. A cooperative tracking algorithm exploiting AOA measurements, obtained by processing beacon packets associated with vehicle-to-vehicle (V2V), in addition to V2I, communications, has been investigated in [18]. In [19], localization using multiple beacons is addressed, and an algorithm is proposed to estimate the angles of arrival (AOAs) in the presence of mutual coupling, ultimately determining the vehicle’s position. The algorithm’s performance is evaluated by analyzing the errors in the estimated AOAs.

Radar systems, a mature technology for remote sensing [20] and surveillance [21], are widely used in a large variety of applications. In particular, radars have gained momentum for automotive applications, see [22] for an overview of state-of-the-art signal processing in automotive radars. Radar-based approaches to self localization have been investigated for both indoor and outdoor scenarios [23,24,25,26]. Ref. [23] proposes a method that can be used in robots equipped with millimeter wave (mmWave) radars to estimate their position by taking advantage of the interference produced by other radars located in the same environment with a well known position. The robot positions are computed using only the AOA of each radar interference. Ref. [24] provides an extensive performance analysis of an off-the-shelf mmWave radar sensor for people localization and tracking. In [26], the vehicle’s position is inferred by association of landmark observations with map landmarks. Millimeter wave radars are a viable and low-cost solution, already available on vehicles, which have the potential to guarantee high accuracy localization at a low computational cost and under adverse weather conditions.

In this paper, we investigate the limiting performance that can be achieved by a positioning algorithm based on radar-based measurements (range and/or azimuth) of landmarks in known positions. The measurements are collected by a radar (or radars) mounted on the vehicle. This positioning idea can be interpreted as a generalization of the traditional approaches based on AOAs and TOAs measurements. In addition, this paper fills the gap of the analysis in [19] where only the Cramér-Rao lower bound (CRLB) in terms of AOA is computed instead of considering the ultimate performance parameters, namely the position of the vehicle.

The paper is organized as follows: the next section introduces the problem from a quantitative standpoint while Section 3 is devoted to the computation of the CRLB expression. Section 4 discusses the impact that range and/or azimuth measurements have on the positioning precision for some choices of the system parameters. Conclusions are provided in Section 5 and Appendix A provides some mathematical details.

### Notation

In the sequel, vectors and matrices are denoted by boldface lower-case and upper-case letters, respectively. The symbols ${\left(\cdot \right)}^{T}$ and ${\left(\cdot \right)}^{-1}$ denote the transpose and the inverse of a matrix, respectively. Regarding the numerical sets, N is the set of natural numbers, R is the set of real numbers, and $R^{N\times M}$ is the Euclidean space of (N×M)-dimensional real matrices (or vectors if M=1). Let $A\in R^{r\times r}$ be a matrix, r∈N: A(i,j) denotes the (i,j)th entry of the matrix ***A***, with 1≤i≤r and 1≤j≤r. The symbol $\frac{\partial f(x)}{\partial x_{i}}$ denotes the first-order partial derivative of the function f(x), $x={\left[x_{1},\ldots ,x_{n}\right]}^{T},$ with respect to the variable $x_{i}$, 1≤i≤n. Similarly, the symbol $\frac{\partial^{2}f\left(x\right)}{\partial x_{j}\partial x_{i}}$ denotes the second-order partial derivative of the function f(x) with respect to the variables $x_{i}$ and $x_{j}$. The acronyms PDF, RV, and IID stand for the probability density function, random variable, and independent and identically distributed, respectively. **0** stands for the null vector/matrix of proper size. Finally, we write $x∼N(m,\sigma^{2})$ if *x* is a Gaussian RV with mean *m* and variance $\sigma^{2}$.

## 2. Problem Formulation

**Figure 1:**
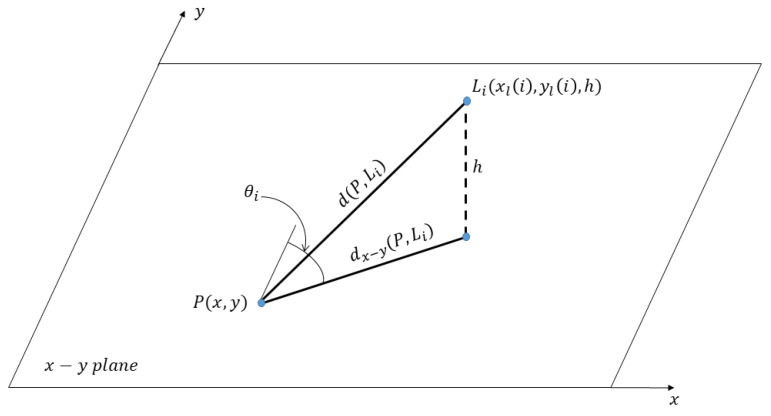
Geometric model of the system with only one landmark. Vehicle is in point *P* and the angle $\theta_{i}$ is measured on the x−y plane.

As previously mentioned, we are concerned with the problem of determining the ultimate performance for vehicle localization and, without loss of generality, we restrict our attention to localization in the planar case. We assume that the vehicle is equipped with a radar and that it is located within a given area somehow delimited by *N* landmarks located at $L_{i}\left(x_{l}\left(i\right),y_{l}\left(i\right)\right),h)$, i=1,…,N, in a given Cartesian reference system; an example with one landmark only is depicted in Figure 1. Of course, when the radar illuminates the environment, other unwanted phenomena emerge, such as unintended scatterers, noisy point clouds, multipath effects, and range ambiguities. In such a case, a problem of data association naturally arises. A possible approach to solve data association and eventually extract the coordinates of the landmarks from radar measurements could be to assume that sparse and noisy point clouds are fed to a constant false alarm rate (CFAR) detector whose output is a map of targets (each characterized by range, azimuth, and velocity). In the following, we only focus on targets with a relative radial velocity that represent the potential landmarks (we assume to know the velocity vector of the vehicle). In addition, we suppose that landmarks transmit their position and that the vehicle is equipped with an array of antennas that can estimate the AoAs of such signals. These AoAs are compared to the estimated azimuthal positions of the detected targets to construct a shorter list of candidate landmarks. The selection can be further refined by exploiting the approximate position of the vehicle that, together with the exact position of each landmark, allows for computing an approximate distance of each landmark from the vehicle. Thus, a comparison of such distance to the selected target ranges measured by the radar will allow for refining the list of detected targets to be associated with each landmark. To avoid ambiguities, it is possible and desirable a judicious displacement of the landmarks that can also be recognized by exploiting their large radar cross section. In this work, we assume that such a task has been already solved and we only investigate the ultimate performance in terms of localization capabilities (using both range and azimuth) by deriving the corresponding CRLB. Finally, notice that our CRLB, based on the simplifying assumption to perfectly recover the landmarks, might be used to discard radar-based positioning methods, whereas, even considering this lower limit, the performance does not fit the application requirements.

Under the above assumptions, we suppose the radar is located at P(x,y) and it is able to measure the range and azimuth of *N* landmarks. As already mentioned, we assume that the heading of the vehicle is known and, for the sake of clarity, that it is aligned with the *y* axis. Assuming correct data association and modeling the estimation errors as zero-mean Gaussian random variables, the measurements of range and azimuth can be modeled as $R_{i}∼N\left(d\left(P,L_{i}\right),\sigma_{r}^{2}\right)$ and $\Theta_{i}∼N\left(\theta_{i},\sigma_{\theta }^{2}\right)$, where $d(P,L_{i})$ indicates the distance of the radar to the *i*th landmark, i.e.,

(1) $$d\left(P,L_{i}\right)=\sqrt{{\left(x-x_{l}\left(i\right)\right)}^{2}+{\left(y-y_{l}\left(i\right)\right)}^{2}+h^{2}}$$

and

(2) $$\theta_{i}=\arcsin\frac{x-x_{l}\left(i\right)}{d_{x-y}\left(P,L_{i}\right)},$$

where

$$d_{x-y}\left(P,L_{i}\right)=\sqrt{{\left(x-x_{l}\left(i\right)\right)}^{2}+{\left(y-y_{l}\left(i\right)\right)}^{2}},$$

is the angle formed by the projection of the line joining *P* and $L_{i}$ onto the x−y plane and the *y* axis, which is positive if measured counterclockwise. We also suppose that $r={\left[R_{1},\cdots ,R_{N}\right]}^{T}$ and $\theta ={\left[\Theta_{1},\cdots ,\Theta_{N}\right]}^{T}$ are independent random vectors. It is important to stress that the Gaussian model for estimation errors of range and azimuth is widely adopted due to its mathematical tractability [27]. However, other more accurate models could be considered; for instance, the von Mises distribution has been used to model AOA measurement errors in direction finding systems [28].

In this paper, we do not propose specific rules to estimate the unknown parameters $x,y,\sigma_{r}^{2},\sigma_{\theta }^{2}$, but we investigate the ultimate achievable performance (i.e., the CRLB).

## 3. Theoretical Bounds on the Achievable Performance

In this section, we derive the CRLB for unbiased estimators of the vehicle position based on *N* landmarks. To this end, we start introducing the loglikelihood function for the problem at hand. Exploiting the assumptions that the entries of the vectors ***r*** and θ are marginally Gaussian and IID, we have the following expression for the joint PDF of ***r*** and θ

$$\begin{array}{cc} f(r,\theta ;x,y,\sigma_{r}^{2},\sigma_{\theta }^{2}) & =\prod_{i=1}^{N}\frac{1}{\sqrt{2\pi \sigma_{\theta }^{2}}}\exp\left[-\frac{1}{2\sigma_{\theta }^{2}}{\left(\Theta_{i}-\theta_{i}\right)}^{2}\right]\prod_{i=1}^{N}\frac{1}{\sqrt{2\pi \sigma_{r}^{2}}}\exp\left[-\frac{1}{2\sigma_{r}^{2}}{\left(R_{i}-d\left(P,L_{i}\right)\right)}^{2}\right] \end{array}$$

with $d(P,L_{i})$ and $\theta_{i}$ given by Equations (1) and (2), respectively. It turns out that the natural logarithm of the likelihood is

(3) $$L\left(x,y,\sigma_{r}^{2},\sigma_{\theta }^{2};r,\theta \right)=L_{r}\left(x,y,\sigma_{r}^{2};r\right)+L_{\theta }\left(x,y,\sigma_{\theta }^{2};\theta \right)$$

with

$$L_{r}\left(x,y,\sigma_{r}^{2};r\right)=C-\frac{N}{2}\log\sigma_{r}^{2}-\frac{1}{2\sigma_{r}^{2}}\sum_{i=1}^{N}{\left(R_{i}-d\left(P,L_{i}\right)\right)}^{2},$$

$$L_{\theta }\left(x,y,\sigma_{\theta }^{2};\theta \right)=C-\frac{N}{2}\log\sigma_{\theta }^{2}-\frac{1}{2\sigma_{\theta }^{2}}\sum_{i=1}^{N}{\left(\Theta_{i}-\theta_{i}\right)}^{2}$$

and $C=-\frac{N}{2}\log\left(2\pi \right)$.

The following theorems contain the expression of the CRLBs for the localization problem at hand.

**Theorem** **1.** *The Fisher information matrix for unbiased estimators of the vector $x={\left[x,y,\sigma_{r}^{2},\sigma_{\theta }^{2}\right]}^{T}$ is given by*

(4) $$J=\frac{1}{\sigma_{r}^{2}}\left[\begin{array}{ccc} J_{r} & 0 & 0\\ 0 & \frac{N}{2\sigma_{r}^{2}} & 0\\ 0 & 0 & 0 \end{array}\right]+\frac{1}{\sigma_{\theta }^{2}}\left[\begin{array}{ccc} J_{\theta } & 0 & 0\\ 0 & 0 & 0\\ 0 & 0 & \frac{N}{2\sigma_{\theta }^{2}} \end{array}\right]$$

*with*

$$J_{r}=\left[\begin{array}{cc} \sum_{i=1}^{N}\frac{{\left(x-x_{l}\left(i\right)\right)}^{2}}{d^{2}\left(P,L_{i}\right)} & \sum_{i=1}^{N}\frac{\left(x-x_{l}\left(i\right)\right)\left(y-y_{l}\left(i\right)\right)}{d^{2}\left(P,L_{i}\right)}\\ \sum_{i=1}^{N}\frac{\left(x-x_{l}\left(i\right)\right)\left(y-y_{l}\left(i\right)\right)}{d^{2}\left(P,L_{i}\right)} & \sum_{i=1}^{N}\frac{{\left(y-y_{l}\left(i\right)\right)}^{2}}{d^{2}\left(P,L_{i}\right)} \end{array}\right]$$

*and*

$$J_{\theta }=\left[\begin{array}{cc} \sum_{i=1}^{N}\frac{{\left(y-y_{l}\left(i\right)\right)}^{2}}{d_{x-y}^{4}\left(P,L_{i}\right)} & -\sum_{i=1}^{N}\frac{\left(x-x_{l}\left(i\right)\right)\left(y-y_{l}\left(i\right)\right)}{d_{x-y}^{4}\left(P,L_{i}\right)}\\ -\sum_{i=1}^{N}\frac{\left(x-x_{l}\left(i\right)\right)\left(y-y_{l}\left(i\right)\right)}{d_{x-y}^{4}\left(P,L_{i}\right)} & \sum_{i=1}^{N}\frac{{\left(x-x_{l}\left(i\right)\right)}^{2}}{d_{x-y}^{4}\left(P,L_{i}\right)} \end{array}\right]$$

**Proof of Theorem** **1.** See Appendix A. □

**Theorem** **2.** *The ultimate mean square (MS) values of the estimation errors on the estimates of x and y are given by the (1,1)th and the (2,2)th entry, respectively, of the inverse of the matrix*

(5) $$J^{\prime }=\frac{1}{\sigma_{r}^{2}}J_{r}+\frac{1}{\sigma_{\theta }^{2}}J_{\theta }.$$

*Obviously, in case localization is performed using measurements of range only, the limiting MS values of the estimation errors on the estimates of x and y are given by the (1,1)th and the (2,2)th entry, respectively, of the inverse of the matrix $\frac{1}{\sigma_{r}^{2}}J_{r}$. Similarly, in case localization is performed using measurements of azimuth only, the reference matrix is $\frac{1}{\sigma_{\theta }^{2}}J_{\theta }$.*

**Proof of Theorem** **2.** The matrix ***J*** is a block diagonal matrix with a first 2 by 2 block and other two blocks consisting of a single element; the first part of the statement is a consequence of the fact that the inverse of ***J*** is the block diagonal matrix formed by the inverses of the blocks of ***J***; the proof of the remaining part of the theorem is straightforward. □

## 4. Illustrative Examples and Discussion

**Figure 2:**
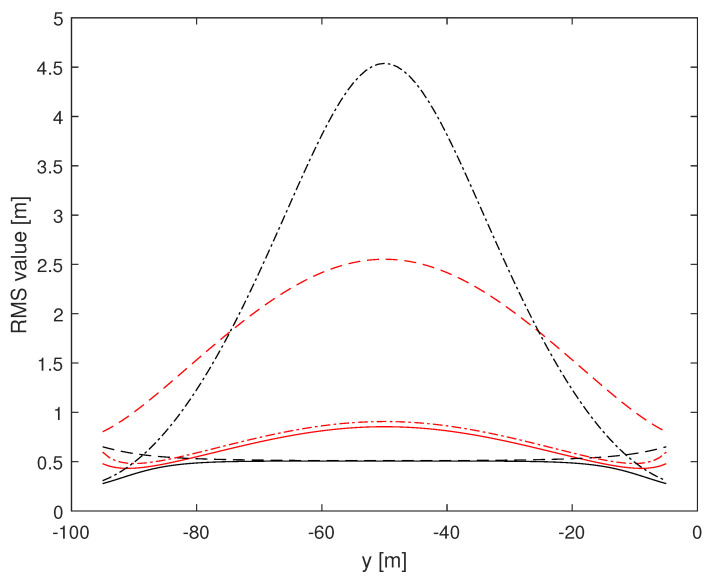
RMS estimation error curves for N=4, d=10 m, $x_{0}=0$ m. (1,1)th: red, (2,2)th: black; solid: range and azimuth measurements, dashed: range measurements only, dash-dotted: azimuth measurements only.

**Figure 3:**
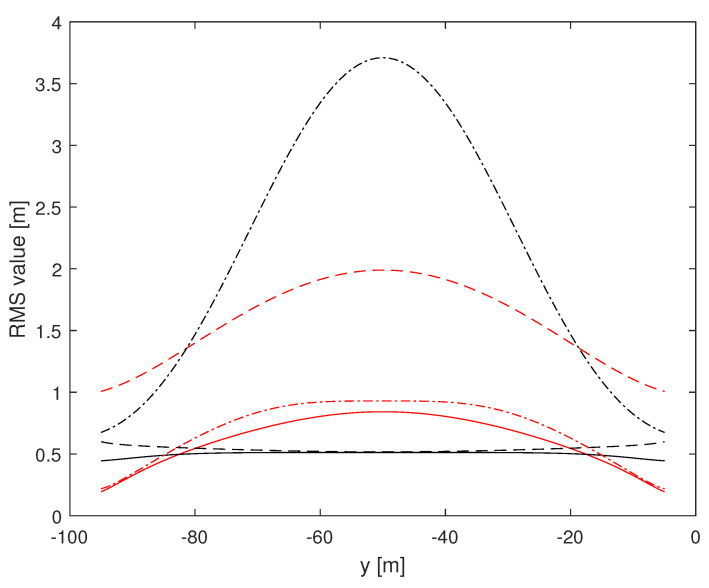
RMS estimation error curves for N=4, d=10 m, $x_{0}=+9$ m. (1,1)th: red, (2,2)th: black; solid: range and azimuth measurements, dashed: range measurements only, dash-dotted: azimuth measurements only.

**Figure 4:**
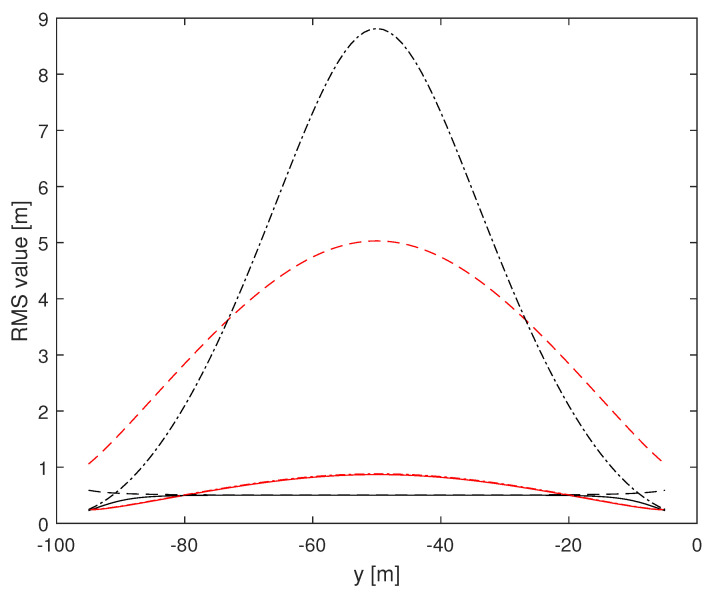
RMS estimation error curves for N=4, d=5 m, $x_{0}=0$ m. (1,1)th: red, (2,2)th: black; solid: range and azimuth measurements, dashed: range measurements only, dash-dotted: azimuth measurements only.

**Figure 5:**
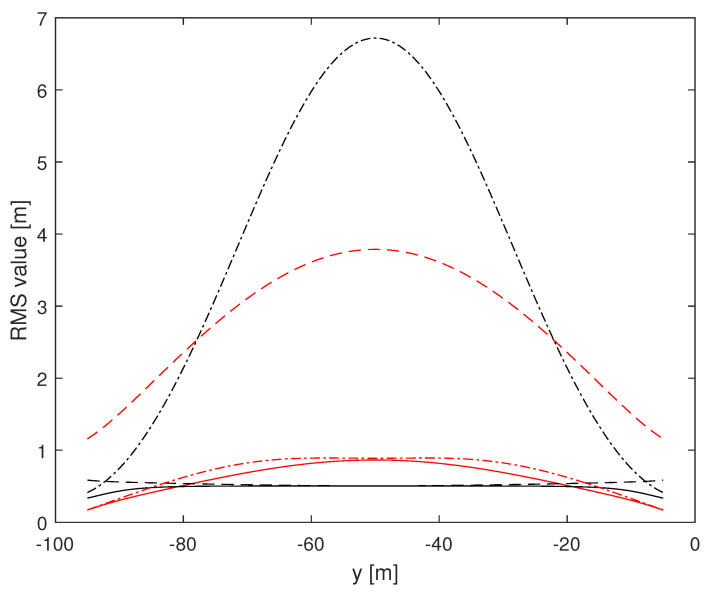
RMS estimation error curves for N=4, d=5 m, $x_{0}=+4.5$ m. (1,1)th: red, (2,2)th: black; solid: range and azimuth measurements, dashed: range measurements only, dash-dotted: azimuth measurements only.

Herein, we investigate the potential performance (i.e., the CRLB) of radar-based self-positioning algorithms based on range and/or azimuth estimates of the landmarks’ positions. To this end, in a first set of examples, we assume N=4 landmarks placed as follows: $L_{1}\left(-d,0,h\right)$, $L_{2}\left(d,0,h\right)$, $L_{3}\left(-d,-100,h\right)$, and $L_{4}\left(d,-100,h\right)$; the height with respect to the radar is h=2.5 m. We also set $\sigma_{r}=1$ m and $\sigma_{\theta }=2{}^{\circ}$. Based on Theorems 1 and 2, we compute the (1,1)th and the (2,2)th entries of the inverse of the matrix $J^{\prime }$ over a uniformly sampled trajectory parallel to the *y* axis, with sampling rate 1 m. More precisely, we consider the positions of a vehicle along a straight line of length 90 m, starting at $x=x_{0}$ m and y=−95 m and ending at $x=x_{0}$ m and y=−5 m. Figure 2 and Figure 3 assume d=10 m, with $x_{0}=0$ m for the former and $x_{0}=9$ m for the latter. Figure 4 and Figure 5 refer instead to d=5 m, $x_{0}=0$ m and $x_{0}=4.5$ m, respectively.

Independent of *d* and $x_{0}$, it is noticed that root mean square (RMS) estimation errors based on measurements of range and azimuth are always less than 1 m on *x* and around 0.5 m on *y*, slightly dependent on *d* and $x_{0}$. A relevant fact is that RMS values on *x* based on azimuth measurements only are close to curves obtained using range and azimuth measurements while RMS estimation errors on *x* based on range measurements only are significantly larger than those based on azimuth measurements. The impact of range and azimuth measurements is reversed considering RMS estimation errors on *y*. However, notice that azimuth measurements may be necessary to correctly associate the landmarks to the measurements. Moreover, observe that combining range and azimuth together improves the overall localization performance with respect to using range or azimuth only. This fact is particularly significant for the *y* axis.

**Figure 6:**
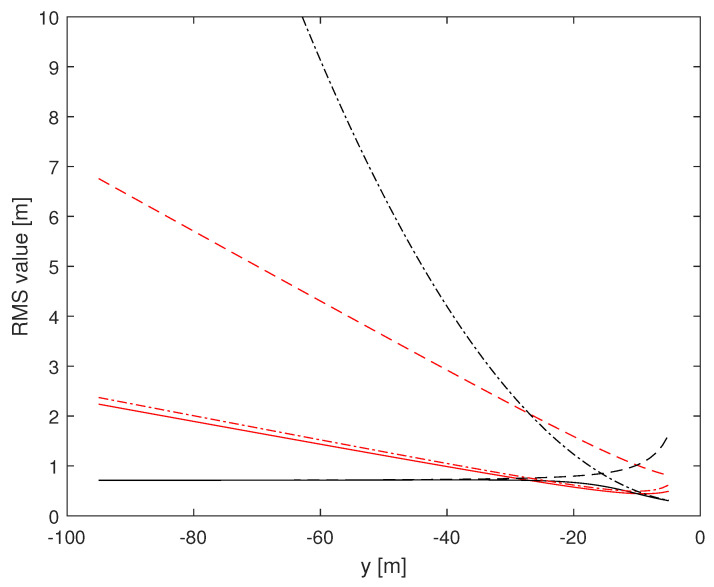
RMS estimation error curves for N=2, d=10 m, $x_{0}=0$ m. (1,1)th: red, (2,2)th: black; solid: range and azimuth measurements, dashed: range measurements only, dash-dotted: azimuth measurements only.

**Figure 7:**
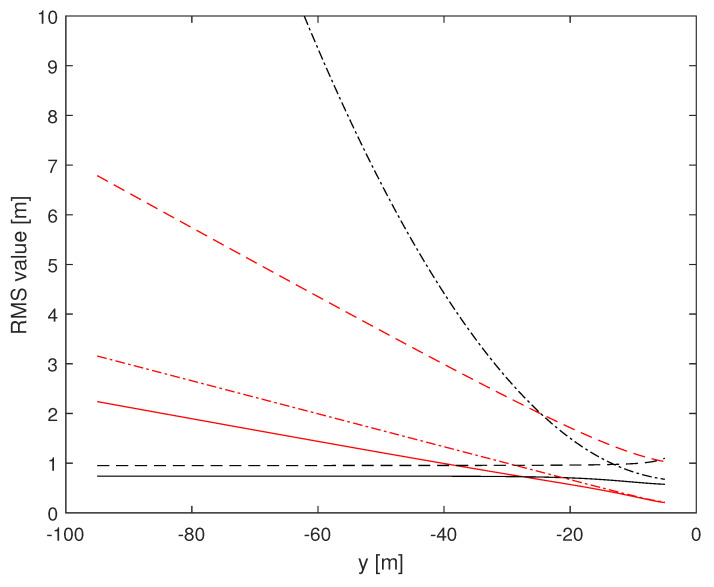
RMS estimation error curves for N=2, d=10 m, $x_{0}=9$ m. (1,1)th: red, (2,2)th: black; solid: range and azimuth measurements, dashed: range measurements only, dash-dotted: azimuth measurements only.

In the sequel, we present a second set of examples with N=2 landmarks. More precisely, we show, in Figure 6 and Figure 7, the performance with $L_{1}\left(-d,0,h\right)$, $L_{2}\left(d,0,h\right)$, d=10 m and h=2.5 m; the vehicle has $x_{0}=0$ m in Figure 6 and $x_{0}=9$ m in Figure 7. As it can be seen, exploiting two landmarks worsens the accuracy of the estimate for the combined range/azimuth measurements, but not dramatically (solid lines).

**Figure 8:**
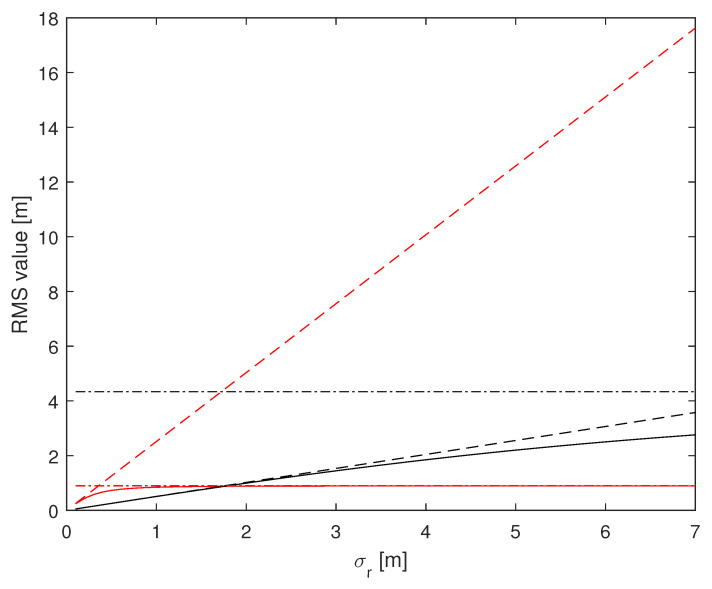
RMS estimation error vs. $\sigma_{r}$ (in m), for N=4, d=10 m, P(0,−45), $\sigma_{\theta }=2{}^{\circ}$. (1,1)th: red, (2,2)th: black; solid: range and azimuth measurements, dashed: range measurements only, dash-dotted: azimuth measurements only.

**Figure 9:**
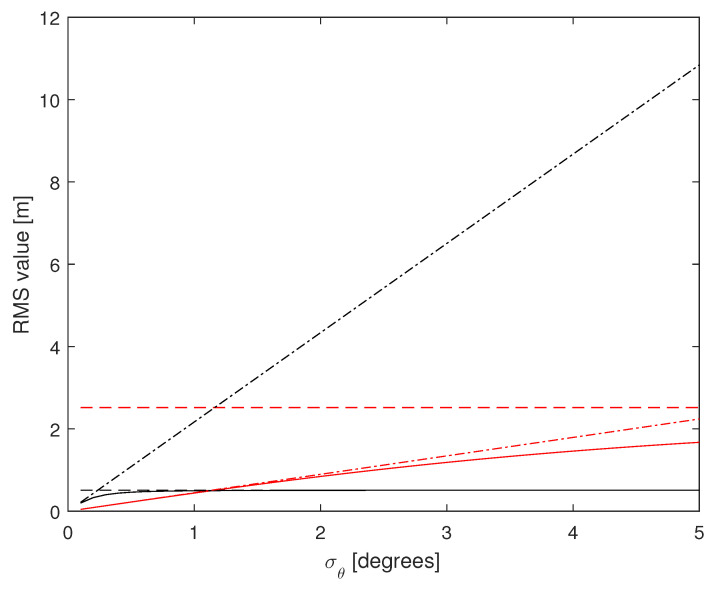
RMS estimation error vs. $\sigma_{\theta }$ (in degrees), for N=4, d=10 m, P(0,−45), $\sigma_{r}=1$ m. (1,1)th: red, (2,2)th: black; solid: range and azimuth measurements, dashed: range measurements only, dash-dotted: azimuth measurements only.

Finally, in a third set of examples, we investigate the trend of the CRLB for varying $\sigma_{r}$ and $\sigma_{\theta }$, in order to show the effect of measurements errors that can arise due to variuos adverse phenomena. The vehicle is located at P(0,−45) and the remaining parameters are those of the first set of examples (Figure 2 and Figure 3). The corresponding RMS values are reported in Figure 8 and Figure 9. Notice from Figure 8 that the RMS value remains under 2 m on *x*, while it tends to increase for *y* (solid and dash-dotted black lines). Of course, estimates based on range-only (dashed lines) are those that suffer most. Conversely, estimates based on azimuth-only (dash-dotted lines) are not affected by variations of $\sigma_{r}$, as it must be expected. The same considerations apply to Figure 9 swapping the roles of range and azimuth.

## 5. Conclusions

In this paper, we investigated the potential of GPS-free positioning schemes and, in particular, we focused on the CRLB of planar localization schemes in which each vehicle estimates its position exploiting range and/or angle measurements of a set of assigned landmarks with a known position. This is motivated by safety applications in scenarios where GPS does not typically provide the required positioning accuracy. The analysis shows that lower bounds on the RMS estimation errors can be less than 1 m on *x* and 0.5 m on *y* using low-cost radar transceivers. Remarkably, the ultimate performance on *x* estimates based on azimuth measurements only are close to what can be obtained using range and azimuth measurements. Regarding *y*, lower bounds obtained using range measurements only were close to what has been computed using range and azimuth measurements.

A possible extension of this work could be the derivation of the CRLB for the case where the measurements are described by a Gaussian Mixture Model, in order to take into account data association errors. We could also focus on the design of techniques to identify the landmarks and eventually to localize the vehicle for the considered scenarios.

## Data Availability

Data are contained within the article.

## Abbreviations

The following abbreviations are used in this manuscript:

AOA	Angle Of Arrival
CFAR	Constant False Alarm Rate
CRLB	Cramér-Rao Lower Bound
GNSS	Global Navigation Satellite System
GPS	Global Positioning System
IID	Independent and Identically Distributed
INS	Intertial Navigation System
ITS	Intelligent Transportation Systems
MS	Mean Square
MUSIC	MUltiple SIgnal Classification
PDF	Probability Density Function
RHS	Right-Hand Side
RMS	Root Mean Square
RSS	Received Signal Strength
RSU	Road Side Unit
RV	Random Variable
TDOA	Time Difference Of Arrival
TOA	Time Of Arrival
V2I	Vehicle-To-Infrastructure
V2V	Vehicle-To-Vehicle
VANET	Vehicular Ad hoc NETwork

## Appendix A

For the sake of clarity, we recall that $x={\left[x,y,\sigma_{r}^{2},\sigma_{\theta }^{2}\right]}^{T}$ and that the loglikelihood, given by Equation (3), is

$$L\left(x,y,\sigma_{r}^{2},\sigma_{\theta }^{2};r,\theta \right)=L_{r}\left(x,y,\sigma_{r}^{2};r\right)+L_{\theta }\left(x,y,\sigma_{\theta }^{2};\theta \right).$$

Thus, it is apparent that

$$\begin{array}{cc} J(3,3) & =-E\left[\frac{\partial^{2}L\left(x;r,\theta \right)}{\partial {\left(\sigma_{r}^{2}\right)}^{2}}\right]=-E\left[\frac{\partial^{2}L_{r}\left(x;r,\theta \right)}{\partial {\left(\sigma_{r}^{2}\right)}^{2}}\right]=-\frac{N}{2\sigma_{r}^{4}}+\frac{1}{\sigma_{r}^{6}}\sum_{i=1}^{N}E\left[{\left(R_{i}-d\left(P,L_{i}\right)\right)}^{2}\right]=\frac{N}{2\sigma_{r}^{4}} \end{array}$$

where we have exploited the statistical characterization of the RVs $R_{i}$. Similarly, we have that

$$\begin{array}{cc} J(4,4) & =-E\left[\frac{\partial^{2}L\left(x;r,\theta \right)}{\partial {\left(\sigma_{\theta }^{2}\right)}^{2}}\right]=-E\left[\frac{\partial^{2}L_{\theta }\left(x;r,\theta \right)}{\partial {\left(\sigma_{\theta }^{2}\right)}^{2}}\right]=-\frac{N}{2\sigma_{\theta }^{4}}+\frac{1}{\sigma_{\theta }^{6}}\sum_{i=1}^{N}E\left[{\left(\Theta_{i}-\theta_{i}\right)}^{2}\right]=\frac{N}{2\sigma_{\theta }^{4}}. \end{array}$$

Obviously, J(3,4) and J(4,3) are zero since

$$\frac{\partial^{2}L\left(x;r,\theta \right)}{\partial \sigma_{\theta }^{2}\partial \sigma_{r}^{2}}=\frac{\partial^{2}L\left(x;r,\theta \right)}{\partial \sigma_{r}^{2}\partial \sigma_{\theta }^{2}}=0.$$

In addition, we have that

$$\begin{array}{cc} \frac{\partial L_{r}\left(x;r,\theta \right)}{\partial x} & =\frac{1}{\sigma_{r}^{2}}\sum_{i=1}^{N}\frac{R_{i}-d\left(P,L_{i}\right)}{d(P,L_{i})}\left(x-x_{l}\left(i\right)\right), \end{array}$$

$$\begin{array}{c} \frac{\partial L_{\theta }\left(x;r,\theta \right)}{\partial x}=\frac{1}{\sigma_{\theta }^{2}}\sum_{i=1}^{N}\left(\Theta_{i}-\arcsin\frac{x-x_{l}\left(i\right)}{d_{x-y}\left(P,L_{i}\right)}\right)\frac{d_{x-y}\left(P,L_{i}\right)-\left(x-x_{l}\left(i\right)\right)\frac{\partial d_{x-y}\left(P,L_{i}\right)}{\partial x}}{d_{x-y}\left(P,L_{i}\right)}\frac{1}{\sqrt{{\left(y-y_{l}\left(i\right)\right)}^{2}}}, \end{array}$$

$$\begin{array}{cc} \frac{\partial L_{r}\left(x;r,\theta \right)}{\partial y} & =\frac{1}{\sigma_{r}^{2}}\sum_{i=1}^{N}\frac{R_{i}-d\left(P,L_{i}\right)}{d(P,L_{i})}\left(y-y_{l}\left(i\right)\right), \end{array}$$

and

$$\begin{array}{c} \frac{\partial L_{\theta }\left(x;r,\theta \right)}{\partial y}=-\frac{1}{\sigma_{\theta }^{2}}\sum_{i=1}^{N}\left(\Theta_{i}-\arcsin\frac{x-x_{l}\left(i\right)}{d_{x-y}\left(P,L_{i}\right)}\right)\frac{x-x_{l}\left(i\right)}{\sqrt{{\left(y-y_{l}\left(i\right)\right)}^{2}}}\frac{1}{d_{x-y}\left(P,L_{i}\right)}\frac{\partial d_{x-y}\left(P,L_{i}\right)}{\partial y}. \end{array}$$

Thus, we observe that

$$\begin{array}{c} J\left(1,3\right)=J\left(3,1\right)=-E\left[\frac{\partial^{2}L_{r}\left(x;r,\theta \right)}{\partial \sigma_{r}^{2}\;\partial x}\right]=\frac{1}{\sigma_{r}^{4}}\sum_{i=1}^{N}\frac{E\left[R_{i}-d\left(P,L_{i}\right)\right]}{d(P,L_{i})}\left(x-x_{l}\left(i\right)\right)=0 \end{array}$$

since $E\left[R_{i}\right]=d\left(P,L_{i}\right)$. Similarly, we have that

$$J\left(1,4\right)=J\left(4,1\right)=-E\left[\frac{\partial^{2}L_{\theta }\left(x;r,\theta \right)}{\partial \sigma_{\theta }^{2}\;\partial x}\right]=0,$$

$$J\left(2,3\right)=J\left(3,2\right)=-E\left[\frac{\partial^{2}L_{r}\left(x;r,\theta \right)}{\partial \sigma_{r}^{2}\;\partial y}\right]=0,$$

and

$$J\left(2,4\right)=J\left(4,2\right)=-E\left[\frac{\partial^{2}L_{\theta }\left(x;r,\theta \right)}{\partial \sigma_{\theta }^{2}\;\partial y}\right]=0.$$

Now observe that

$$\begin{array}{c} \frac{\partial^{2}L_{r}\left(x;r,\theta \right)}{\partial x^{2}}=\frac{1}{\sigma_{r}^{2}}\sum_{i=1}^{N}\left[-\frac{R_{i}{\left(x-x_{l}\left(i\right)\right)}^{2}}{d^{3}\left(P,L_{i}\right)}+\frac{R_{i}-d\left(P,L_{i}\right)}{d(P,L_{i})}\right] \end{array}$$

and hence

(A1) $$-E\left[\frac{\partial^{2}L_{r}\left(x;r,\theta \right)}{\partial x^{2}}\right]=\frac{1}{\sigma_{r}^{2}}\sum_{i=1}^{N}\frac{{\left(x-x_{l}\left(i\right)\right)}^{2}}{d^{2}\left(P,L_{i}\right)}.$$

Similarly, we have

(A2) $$-E\left[\frac{\partial^{2}L_{r}\left(x;r,\theta \right)}{\partial y^{2}}\right]=\frac{1}{\sigma_{r}^{2}}\sum_{i=1}^{N}\frac{{\left(y-y_{l}\left(i\right)\right)}^{2}}{d^{2}\left(P,L_{i}\right)}$$

and

(A3) $$-E\left[\frac{\partial^{2}L_{r}\left(x;r,\theta \right)}{\partial y\partial x}\right]=\frac{1}{\sigma_{r}^{2}}\sum_{i=1}^{N}\frac{\left(x-x_{l}\left(i\right)\right)\left(y-y_{l}\left(i\right)\right)}{d^{2}\left(P,L_{i}\right)}.$$

Now notice that $\frac{\partial L_{\theta }\left(x;r,\theta \right)}{\partial x}$ can be written as

$$\begin{array}{c} \frac{\partial L_{\theta }\left(x;r,\theta \right)}{\partial x}=\frac{1}{\sigma_{\theta }^{2}}\sum_{i=1}^{N}\left(\Theta_{i}-\arcsin\frac{x-x_{l}\left(i\right)}{d_{x-y}\left(P,L_{i}\right)}\right)g_{1,i}\left(x,y\right), \end{array}$$

where

$$g_{1,i}\left(x,y\right)=\frac{d_{x-y}\left(P,L_{i}\right)-\left(x-x_{l}\left(i\right)\right)\frac{\partial d_{x-y}\left(P,L_{i}\right)}{\partial x}}{d_{x-y}\left(P,L_{i}\right)}\frac{1}{\sqrt{{\left(y-y_{l}\left(i\right)\right)}^{2}}}$$

and hence

$$\begin{array}{cc} \frac{\partial^{2}L_{\theta }\left(x;r,\theta \right)}{\partial x^{2}} & =-\frac{1}{\sigma_{\theta }^{2}}\sum_{i=1}^{N}\frac{1}{{\left(y-y_{l}\left(i\right)\right)}^{2}}{\left(\frac{d_{x-y}\left(P,L_{i}\right)-\left(x-x_{l}\left(i\right)\right)\frac{\partial d_{x-y}\left(P,L_{i}\right)}{\partial x}}{d_{x-y}\left(P,L_{i}\right)}\right)}^{2}\\ & +\frac{1}{\sigma_{\theta }^{2}}\sum_{i=1}^{N}\left(\Theta_{i}-\arcsin\frac{x-x_{l}\left(i\right)}{d_{x-y}\left(P,L_{i}\right)}\right)\frac{\partial g_{1,i}\left(x,y\right)}{\partial x}. \end{array}$$

Since the second addend has zero mean, it turns out that

(A4) $$-E\left[\frac{\partial^{2}L_{\theta }\left(x;r,\theta \right)}{\partial x^{2}}\right]=\frac{1}{\sigma_{\theta }^{2}}\sum_{i=1}^{N}\frac{{\left(y-y_{l}\left(i\right)\right)}^{2}}{d_{x-y}^{4}\left(P,L_{i}\right)},$$

where we used

$$\frac{\partial d_{x-y}\left(P,L_{i}\right)}{\partial x}=\frac{x-x_{l}\left(i\right)}{d_{x-y}\left(P,L_{i}\right)}.$$

Similarly, we write

$$\begin{array}{cc} \frac{\partial L_{\theta }\left(x;r,\theta \right)}{\partial y} & =-\frac{1}{\sigma_{\theta }^{2}}\sum_{i=1}^{N}\left(\Theta_{i}-\arcsin\frac{x-x_{l}\left(i\right)}{d_{x-y}\left(P,L_{i}\right)}\right)g_{2,i}\left(x,y\right), \end{array}$$

with

$$g_{2,i}\left(x,y\right)=\frac{x-x_{l}\left(i\right)}{\sqrt{{\left(y-y_{l}\left(i\right)\right)}^{2}}}\frac{1}{d_{x-y}\left(P,L_{i}\right)}\frac{\partial d_{x-y}\left(P,L_{i}\right)}{\partial y}.$$

Thus, computing the second-order derivative of $L_{\theta }\left(x;r,\theta \right)$ with respect to *y*, yields

$$\begin{array}{cc} \frac{\partial^{2}L_{\theta }\left(x;r,\theta \right)}{\partial y^{2}} & =-\frac{1}{\sigma_{\theta }^{2}}\sum_{i=1}^{N}\frac{d_{x-y}\left(P,L_{i}\right)}{\sqrt{{\left(y-y_{l}\left(i\right)\right)}^{2}}}\frac{x-x_{l}\left(i\right)}{d_{x-y}^{2}\left(P,L_{i}\right)}\frac{\partial d_{x-y}\left(P,L_{i}\right)}{\partial y}g_{2,i}\left(x,y\right)-\frac{1}{\sigma_{\theta }^{2}}\sum_{i=1}^{N}\left(\Theta_{i}-\arcsin\frac{x-x_{l}\left(i\right)}{d_{x-y}\left(P,L_{i}\right)}\right)\frac{\partial g_{2,i}\left(x,y\right)}{\partial y} \end{array}$$

and, since the second addend has zero mean, it follows that

(A5) $$-E\left[\frac{\partial^{2}L_{\theta }\left(x;r,\theta \right)}{\partial y^{2}}\right]=\frac{1}{\sigma_{\theta }^{2}}\sum_{i=1}^{N}\frac{{\left(x-x_{l}\left(i\right)\right)}^{2}}{d_{x-y}^{4}\left(P,L_{i}\right)},$$

where we have used

$$\frac{\partial d_{x-y}\left(P,L_{i}\right)}{\partial y}=\frac{y-y_{l}\left(i\right)}{d_{x-y}\left(P,L_{i}\right)}.$$

Similarly, we prove that

(A6) $$-E\left[\frac{\partial^{2}L_{\theta }\left(x;r,\theta \right)}{\partial y\partial x}\right]=-\frac{1}{\sigma_{\theta }^{2}}\sum_{i=1}^{N}\frac{\left(x-x_{l}\left(i\right)\right)\left(y-y_{l}\left(i\right)\right)}{d_{x-y}^{4}\left(P,L_{i}\right)}.$$

Finally, observe that J(1,1) is the sum of the right-hand side (RHS) of Equations (A1) and (A4) and, similarly, J(2,2) is the sum of the RHS of Equations (A2) and (A5). Finally, J(1,2)=J(2,1) is the sum of the RHS of Equations (A3) and (A6) and Theorem 1 is thus proved.
